# Prevalence of Malnutrition and Its Related Factors among Pre-School (2–6 Years) Children in the Neighborhood of Chahestaneha, Bandar-Abbas, Iran

**Published:** 2019-10

**Authors:** Kabir Ozigi ABDULLAHI, Fatima Mahmud MUHAMMAD, Kourosh HOLAKOUIE-NAIENI, Ahmad KHOSRAVI, Fahad Saqib LODHI, Ahang Abdullah KAREEM, Aram Salih Mohammed Amin KAMALI, Hasnain SABAHAT, Sami Najmaddin SAEED, Mohammad Ali HOSSEINI

**Affiliations:** 1. Department of Medico-Surgical Nursing, School of Nursing & Midwifery, Tehran University of Medical Sciences, Tehran, Iran; 2. Department of Epidemiology and Biostatistics, School of Public Health, Tehran University of Medical Sciences, Tehran, Iran

## Dear Editor-in-Chief

Good nutrition is that adequate and well balanced one containing essential nutrient in an appropriate proportion (1). Malnutrition is an imbalanced nutritional status of an individual, insufficient intake of nutrients, absorption and sometimes of pathological origin resulting to illnesses of varying degrees of severity and diverse clinical manifestations, and deaths in extreme cases especially among young children in the developing countries. Indicators of malnutrition are wasting, under-weight and stunting. Wasting is represented by the weight of an individual for their age as its reflected in their body size, underweight portrays the height of an individual for their age, it reflects the achieved linear growth while stunting represent the height of children as shorter than expected for their age and gender group with reference to their population due to either past chronic nutritional deficiency or pathological state (2, 3). Globally, one out of every three pre-school children are malnourished, there is an estimation of 165 million under five years children were underweight, 155 million are stunted and 52 million are wasted, while 41 million are either overweight or obese (4).

With its peculiar nature, these influence the researcher after completing a community assessment on the study area (Chahestaneha in Bandar Abbas, the Hormozgan Province, Iran) to carry out this study in Jan 2015. Considering the community strength, resources, and their attending problems, a cross-sectional study was conducted to determine the prevalence of malnutrition among children ages 2–6 yr of Chahestaneha area of Bandar Abbas district. The sample consisted of all households with children in the age group between 2–6 yr were included and all other children outside this ages limit were excluded and also those families who refused to participate voluntarily were also excluded in the study. Cluster sampling was used to divide the community into 12 clusters.

Each unit of the cluster is composed of at least 15 households, where informed consent was secured from the head of the household and parents of the selected children. Interview from the parent using standardized structured questionnaires was used to assess the knowledge, attitude, and practice of the parents regarding nutrition in the study area and at the same time, the anthropometric measurements of the participants were taken. All questionnaires were checked for completeness, accuracy, and consistency, the data so collected was entered into SPSS ver. 22 (Chicago, IL, USA) and the anthropometric measurement were analyzed using Epi info software ver. 6, then Principal Component Analysis (PCA) in which all the variables regarding socio-economic status of the families of the participant was reduced to one data set variable using Z-score Table 1.

**Table 1:** Z-score categorization of malnutrition

***Nutrition***	***Status classification***	***Z-score (SD)***
Wasting (Weight to age)	Normal	≤ −1
	Slight	−1 < Z ≤ − 2
	Medium	−2 < Z ≤ −3
	Acute	< − 3
Underweight (Height for Age)	Normal	≤ −1
	Slight	−1 < Z ≤ − 2
	Medium	−2 < Z ≤ −3
	Acute	< − 3
Stunting (Weight to Height)	Normal	≤ −1
	Slight	−1 < Z ≤ − 2
	Medium	−2 < Z ≤ −3
	Acute	< − 3

The study was done on 290 children within the ages between 2–6 yr of the study area out of which 36.0% of the children were underweight, 45.1% were stunted and 55.2% wasted and the prevalence of underweight had a significant relationship with weight of the child at birth (*P-*value=0.034). Moreover, the prevalence of stunting had a significant relationship with mother's occupation (*P-*value=0.048) (Table 2).

**Table 2:** Comparison of the prevalence of malnutrition with the weight of the child at birth and the mother's occupation

***Variables***	***Xtics***	***Underweight***		***Stunting***		***Wasting***	
		***Underweight (%)***	***Normal (%)***	***Stunted (%)***	***Normal (%)***	***Wasted (%)***	***Normal (%)***
Weight at birth	Low	31(46.3)	36(53.7)	28(41.8)	39(58.2)	41(61.2)	26(38.8)
	Normal	67(32.1)	142(67.9)	100(47.8)	109(52.2)	116(55.0)	95(45.0)
	*P*-value*	*P*= 0.034*		*P*= 0.387		*P*= 0.371	
Mother's Occupation	House wife	149(54.4)	125(45.6)	120(44.1)	152(55.9)	149(54.4)	125(45.6)
	Simple worker	3(50.0)	3(50.0)	2(33.3)	4(66.7)	3(50.0)	3(50.0)
	Govt. employee	9(90.0)	1(10.0)	9(90.0)	1(10.0)	9(90.0)	1(10.0)
	*P*-value*	*P*= 0.196)		*P*= 0.048*		*P*= 0.196	

* Chi-square test, *P* is significant at the level below α =0.05

Overall, 36% of the participant were under-weight, this is similar with the study conducted in the South Khorasan, Iran (47.8%) (2), Tanzania (5.74%) (5) and Ghana (13.8%) (6) (*P*=0.034) while 45.1% of the children were stunted, similar result was obtained in South Khorasan, Iran (45%) (2), Afghanistan (35%) (7), but in contrast with these studies with lower result in Tanzania (8.34%) (5). Moreover, the prevalence of stunting was significantly associated with the mother's occupation. 55.2% wasted which is similar to the following study in South Khorasan (32.2%) (2) in contrast to the study with 1.41% in Tanzania (5) and Ghana 8.9% (6).

This high prevalence of malnutrition may be due to poor or inadequate weaning diet.
